# Development of Local Natural Orbital Arbitrary Order Coupled Cluster Methods and Assessment through Connected Quadruples

**DOI:** 10.1021/acs.jpca.6c00607

**Published:** 2026-04-08

**Authors:** Vladimir Fishman, Balázs D. Lőrincz, Emmanouil Semidalas, Aditya Barman, Jan M. L. Martin, Péter R. Nagy, Mihály Kállay

**Affiliations:** † Department of Molecular Chemistry and Materials Science, 34976 Weizmann Institute of Science, Reḥovot 7610001, Israel; ‡ Department of Physical Chemistry and Materials Science, Faculty of Chemical Technology and Biotechnology, 61810 Budapest University of Technology and Economics, Műegyetem Rkp. 3, Budapest H-1111, Hungary; § HUN-REN−BME Quantum Chemistry Research Group, Műegyetem Rkp. 3, Budapest H-1111, Hungary; ∥ MTA−BME Lendület Quantum Chemistry Research Group, Műegyetem Rkp. 3, Budapest H-1111, Hungary; ⊥ Department of Chemistry, National and Kapodistrian University of Athens, Panepistimiopolis Zografou, Athens 15772, Greece

## Abstract

We present the development of local natural orbital (LNO)-based arbitrary order coupled cluster (CC) methods and a rigorous assessment of the beyond CCSD­(T)-level correlation through LNO-CCSDTQ. Both the closed- and open-shell implementations inherit from our LNO family of methods the asymptotically linear-scaling framework, support for point group symmetry, multilevel embedding, as well as greatly reduced memory and disk use. The accuracy of LNO approximations and basis set completeness is benchmarked for thermochemistry and noncovalent interactions through CCSDTQ. Even for complicated atomizations and barrier heights, the CCSDT–CCSD­(T), (Q), and especially the CCSDT­(Q)–CCSD­(T) contributions are obtained reliably within 85–95% relative and 0.05–0.1 kcal/mol absolute accuracy, already with the default LNO threshold set. Tightening LNO settings generally improves this performance, useful when aiming at high relative accuracy in even smaller effects, such as in noncovalent interactions or when pursuing the basis set limit of post-CCSD­(T) atomization contributions. Thus, the LNO approximation greatly expands the reach of post-CCSD­(T) methods, especially beyond a few atoms and small (double-ζ) basis sets, i.e., above ca. 100 orbitals. For example, via our multilevel approach, we report an unprecedented CCSDT­(Q)–CCSD­(T) reaction energy correction computation for a real-life enzyme reaction. Thus, LNO-based higher-order CC methods enable thermochemistry protocols aiming at kJ/mol accuracy for practical, 3D molecular systems that are much larger than previously accessible.

## Graphical abstract

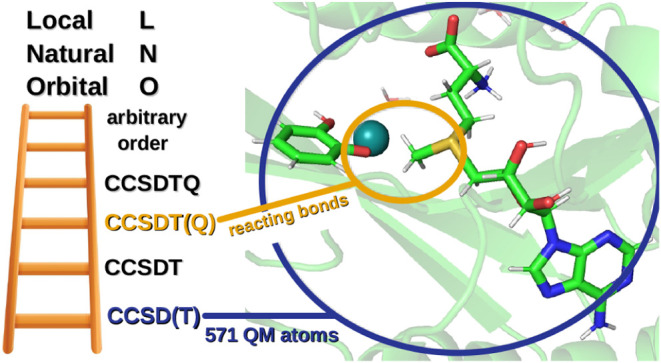

## Introduction

High accuracy in quantum chemical calculations is crucial for reliable predictions, such as in thermochemistry, reaction kinetics, and noncovalent interactions, among other properties. The coupled cluster (CC) family of methods is one of the most powerful for this purpose, especially due to its systematic improvability via including single (S), double (D), triple (T), quadruple (Q), etc. excitations. Here, we employ the arbitrary order CC approach developed by one of us,^1^ focusing mostly on the CCSDT and CCSDTQ levels, as well as when triple or quadruple excitations are treated perturbatively in the CCSD­(T)^2–4^ and CCSDT­(Q)^5,6^ approaches. The CCSD­(T) method has been considered as the gold standard of quantum chemistry, particularly for well-behaved single-reference systems, while higher orders of the CC hierarchy provide systematically better accuracy and a wider applicability range for more complicated electronic structures.^7,8^ Post-CCSD­(T) methods also crucially contribute to the success of high-accuracy thermochemistry protocols.^9–14^ Nevertheless, applications to larger systems are hampered by their steep computational scaling, which is gradually increasing from O­(*N* ^7^) for CCSD­(T) up to O­(*N* ^10^) for CCSDTQ.

By exploiting the short-range nature of electron correlation, local correlation CC methods provide a solution to reduce the scaling potentially up to asymptotically linear, at least for very large systems. Among the many flavors of local correlation approaches,^15–35^ here we employ the local natural orbital (LNO) method developed by Kállay, Nagy, and coworkers^36–45^ in the Mrcc program suite.^46–48^ While early developments of high-order local CC in Mrcc were reported long ago,^36^ with advancements in combination with the updated closed-shell LNO methodology,^40^ our main focus previously was on the optimization, validation, and application of LNO methods through LNO-CCSD­(T).^39–42,45^ Very recently, also the domain-based local pair NO (DLPNO) approach of Neese and coworkers^21–23^ has been extended to the CCSDT,^49^ CCSDT­(Q),^50^ and CCSDTQ^51^ levels by Jiang, Schaefer, Turney, and coworkers. The accuracy benchmarks of refs 49. and^50^ are very promising, while the steep scaling of canonical CCSDT and CCSDT­(Q) only allowed for calculations for somewhat special cases, up to medium-sized, low-dimensional systems, such as alkane chains and water molecular clusters. Therefore, further development and benchmarking are still required for higher-order local CC methods in general.

Here, we report an implementation of LNO-CC up to arbitrary order iterative CC as well as for CCSDT­(Q), suitable for both closed- and open-shell systems. The higher-order CC implementation also includes current LNO features reported so far up to LNO-CCSD­(T). These features include the utilization of general (including non-Abelian) point group symmetry, multilevel correlation embedding,^52,53^ and asymptotically linear-scaling cost and constant memory and disk requirement for the LNO construction.^40–42,45^ The LNO approximations are also arranged in a systematically improvable hierarchy that allows the control of the overall LNO accuracy with user-friendly, predefined threshold combinations.^41,45^ Moreover, these LNO threshold settings enable extrapolations toward the canonical CC results, which also provides a robust LNO error estimate.^41,45^

Furthermore, we report a hitherto missing benchmark of the LNO approximation for post-CCSD­(T) corrections. Although the LNO approach is thoroughly assessed up to the CCSD­(T) level, extending this validation beyond (T) is crucial for practical applications. The most straightforward way is comparison against a canonical CC reference, which has been reported for LNO-CCSD­(T) for more than 1,000 data points, covering common energy difference properties from 14 benchmark data sets (see review in ref 45). For example, Nagy, Kállay and coworkers reported highly competitive mean absolute error (MAE) metrics of 0.1–0.25 kcal·mol^–1^ already with the default LNO settings for medium-size systems involving organic reactions,^41^ noncovalent interactions,^54^ as well as open-shell systems including radical reactions, ionization potentials, and spin-state gaps.^42^ Notably, any local correlation method, including LNO, has increased deviations for larger molecules and more complicated electronic structures. For example, MAEs of 0.3–0.5 kcal·mol^–1^ are found even for the very complicated tests collected in ref 45, including the largest possible molecules where canonical CCSD­(T) is still possible.

In addition, the Martin group has been particularly active in benchmarking local correlation methods, where LNO-CCSD­(T) was found to be highly competitive. These works include rare benchmarks for large systems, such as noncovalent interactions^55,56^ for the standard S66 ^57^ and S66 × 8 ^58^ data sets up to 34 atoms, conformation energies up to 38 atoms,^59^ reactions and transition states (TSs) involving extended and complicated polypyrroles up to 67 atoms,^60^ as well as organometallic reactions and barriers between practical transition metal complexes of up to 65 atoms,^61^ such as those in the MOBH35 database^62,63^ as well as Ru catalysts.^64^ Other independent and complementary benchmarks also report the high accuracy of LNO-CCSD­(T) for processes involving atomization,^65^ isomerization,^66^ and ionic interactions.^67^ These tests and numerous other demonstrations also for large systems of 50–150 atoms show that LNO-CCSD­(T) results converge systematically toward the local approximation free and near the complete basis set (CBS) limits, as the LNO thresholds are tightened and the basis set size is increased.^41,45^ Moreover, efficiency benchmarks reveal highly competitive performance and the unique ability of LNO-CCSD­(T) to go beyond 300 atoms at the CBS limit, scaling even up to 1000 atoms for proteins with quadruple-ζ basis sets.^41,45^

Building on these foundations, in this paper, we also investigate the accuracy of the LNO-based post-CCSD­(T) models against canonical references. Detailed analysis up to CCSDTQ is given for bond separation energies (BSE)^68^ and total atomization energies (TAE) of 160 molecules from the W4-17 data set.^11^ The somewhat larger and more complicated reactions of the BH28 ^69^ set, as well as the Cope rearrangement of semibullvalene, are used to assess the accuracy of barrier heights up to LNO-CCSDT­(Q). Furthermore, for a subset of diatomic and triatomic closed-shell molecules (41 species in total), we examine the basis set superposition error (BSSE) effect on the LNO-based post-CCSD­(T) terms. Next, we consider the performance of LNO-CCSDT and LNO-CCSDT­(Q) for noncovalent interactions in the 1.00*r*_*e*_ slice of the S66 × 8 data set.^58^ Then, to assess the efficiency, we determine the crossover point at which the gentler scaling LNO-CCSDT and LNO-CCSDT­(Q) methods start to outperform their canonical counterparts, as well as their shared memory parallel scaling. Finally, to demonstrate the unlocked possibilities, we report a previously unfeasible, multilevel LNO-based CCSDT­(Q)–CCSD­(T) correction computation to a real-world enzyme reaction.

## Theory and Implementation

The LNO family of methods includes, at the M1 level of theory, MP2,^38,43^ random phase approximation (RPA),^70^ (as well as the corresponding spin-component scaled and double-hybrid DFT methods utilizing local MP2 or RPA), CCSD­(T),^37,39–42,44,45^ while here we detail the LNO-based general order CC methods.^36,41^ All of these correlation energies can be expressed in terms of the individual correlation energy contributions of localized molecular orbitals (LMOs, *I*, *J*) as

1 $$E^{\mathrm{LNO}-M1}=\underset{I}{\sum }\delta E_{I}=\underset{I}{\sum }\left[\delta E_{I}^{\mathrm{LNO}-M1}+\Delta E_{I}^{\mathrm{MP}2}+\frac{1}{2}\overset{\mathrm{distant}\;\mathrm{p.}}{\underset{J}{\sum }}\delta E_{IJ}^{\mathrm{MP}2}\right]$$

where, for instance, LNO-CCSDT­(Q) is obtained as M1 = CCSDT­(Q). Without any local and NO approximations, the sum of the first, $\delta E_{I}^{\mathrm{LNO}-M1}$ orbital specific correlation energy contributions recover the exact M1 level correlation energy, e.g., the M1 = CCSDT­(Q) result, and the last two correction terms of eq 1 vanish. $\Delta E_{I}^{\mathrm{MP}2}$ is an MP2-level correction for the truncation of the LNO-space within the domain constructed for LMO *I*. For larger molecules, the correlation energy contribution of distant LMO pairs is included via approximate MP2 expressions $\left(\delta E_{IJ}^{\mathrm{MP}2}\right)$ .^38,40^ While this part is mostly inoperative for the relatively small systems studied, e.g., in the W4-17 ^11^ data set here, the general benefit is that the more costly $\delta E_{I}^{\mathrm{LNO}-M1}$ and $\Delta E_{I}^{\mathrm{MP}2}$ terms of eq 1 are only evaluated for an asymptotically linear-scaling list of strongly interacting LMO pairs. As the same pair and domain approximations are used for our LMP2 and LNO-CCSD­(T) approaches as for our LNO-based general order CC methods here, we refer to those works for extensive details.^38,40–43^ More importantly, the major acceleration for LNO-based post-CCSD­(T) computations comes from the natural orbital-based compression of both the occupied and virtual orbital spaces of the M1 computation, which is a unique advantage of our LNO approach.^41,42,45^ Compressing both spaces with LNOs is especially beneficial considering the resulting up to *O*(*N* ^8^)-, *O*(*N* ^9^)-, and *O*(*N* ^10^)-scaling cost-reduction for LNO-CCSDT, LNO-CCSDT­(Q), and LNO-CCSDTQ, respectively. In comparison, PNO methods compress only the virtual MO space. On top of this, both LNO and DLPNO methods implement occupied orbital pair screening, which in principle can lead to asymptotically linear scaling. For completeness, we note that linear scaling can only be expected for large molecules that are at present out of reach with post-CCSD­(T) methods.

Our approach closely follows the LNO approximations reported for our highly optimized closed- and open-shell LNO-CCSD­(T) methods,^40–42^ so here we only collect the aspects relevant for post-CCSD­(T). For compatibility, the LNO orbital spaces used for post-CCSD­(T) are constructed in the same way as for LNO-CCSD­(T).^40–42^ However, in these LNO bases, instead of our hand-optimized CCSD­(T) codes,^71,72^ the arbitrary order^1^ and higher-order perturbatively corrected^6^ CC codes are executed.^36,41,42^ In the LNO context, our hand-optimized codes up to CCSD­(T) are available in Mrcc via the ccprog=ccsd keyword, while the general-order CC code via ccprog=mrcc supports CC methods starting from CCSD to all arbitrary order iterative CC methods, as well as CCSD­(T) and CCSDT­(Q). Up to CCSD­(T), the efficiency, parallelization, as well as the memory- and disk-economic implementation of the hand-optimized code is clearly preferred, and this is set as the default. Above the CCSD­(T) level, ccprog=mrcc is the only available option. Thus, CCSD and CCSD­(T) are available both via the hand-optimized ccprog=ccsd and the code-generated ccprog=mrcc options. However, unfortunately not 100% of the features available through LNO-CCSD­(T) via ccprog=ccsd are implemented fully consistently for the LNO-based post-CCSD­(T) methods. Consequently, when evaluating LNO-based post-CCSD­(T) corrections, such as CCSDT­(Q)–CCSD­(T) or CCSDT–CCSD­(T) terms of common interest, the consistent use of ccprog=mrcc is recommended for both LNO-CCSD­(T) and the higher-order LNO-CC parts. The thus obtained LNO-based post-CCSD­(T) correction can then be combined with the much more efficient LNO-CCSD­(T) energy, using, e.g., a larger basis set and/or better converged LNO settings up to the LNO-CCSD­(T) level, in the fashion of focal point approaches.

Let us turn briefly to the differences compared to the previous approaches for LNO-CCSD­(T). The set of LNO approximations also employs the so-called natural auxiliary function (NAF) approach^73^ to compress the auxiliary function space used for the density fitting (DF) approximation. The NAF compression rate is usually very high, as the NAFs are optimized for the compressed occupied and virtual LNO spaces. Both of the LNO-CC methods via ccprog=ccsd and ccprog=mrcc include an MP2 level correction for the NAF compression as part of the $\Delta E_{I}^{\mathrm{MP}2}$ term of eq 1. The difference is that for the LNO-CCSD­(T) energies evaluated via ccprog=ccsd an additional NAF correction^73^ is implemented, which employs the four-center electron repulsion integrals assembled in the complete DF basis, i.e., before NAF compression. This second, somewhat less important, correction is not available with the arbitrary order CC methods. Thus, ccprog=ccsd and ccprog=mrcc results for LNO-CCSD and LNO-CCSD­(T) are not completely identical, which deviation cancels well from energy differences in practice.

To explain a second point, let us briefly recall our LNO approach for treating open-shell systems.^42,43^ The key detail is that the LNO correlation energy computation is performed in a restricted open-shell basis to save resources. It is important to highlight that both restricted open-shell as well as unrestricted [Hartree–Fock (HF) or Kohn–Sham (KS)] reference determinant computations are supported and compatible with open-shell LNO-CC in Mrcc. However, when using an unrestricted reference, the self-consistent unrestricted orbitals are basically projected to a restricted open-shell form (cf. quasi-restricted orbitals, QROs, in ref 43.), and then the localization and correlation calculation is performed with this (quasi-) restricted open-shell orbital space. This treatment, developed for LNO-CCSD­(T), is also inherited by the higher-order open-shell LNO-CC methods. The difference originates from the treatment of the terms in (T) and (Q) that depend on the occupied-virtual block of the Fockian, emerging only due to the nonself-consistent nature of the reference determinant. The term of (T) in question is included in our open-shell LNO-CCSD­(T) method evaluated via the hand-optimized ccprog=ccsd, while we employ the approximation of neglecting these relatively small terms in the LNO-based perturbative corrections methods, when using ccprog=mrcc. All in all, as we shall demonstrate in the Results and Discussion Section, this approximation works very well.

Finally, let us summarize additional unique features of our LNO implementation that are also made available for the post-CCSD­(T) levels. Namely, point group symmetry is supported, as detailed in ref 41. The idea, briefly, is that if a symmetry operation transforms LMO *I* to LMO *J*, then their δ*E*_*I*_ and δ*E*_*J*_ correlation energy contributions are the same. Thus, we compute the equivalent contributions only once, yielding a speedup comparable to the number of symmetry elements of the point group, including also support for non-Abelian point groups. Moreover, the form of the LNO energy expression of eq 1 also enables multilevel correlations approaches.^52^ Namely, the more important, e.g., chemically active LMOs can be treated at a higher level (HL) of theory, while the correlation from the orbitals localized on the chemical environment can be included cost-effectively using a lower level (LL) model. The assignment of LMOs to HL and LL is fully automated via robust algorithms and requires only the list of atoms as the definition of the active subsystem.^52^ Moreover, Mrcc implements a wide range of multilevel methods both for closed- and open-shell systems.^52^ Two levels can be selected at the LNO-based correlated level, leading to the following multilevel LNO-HL-in-LNO-LL correlation energy:

2 $$E^{\mathrm{LNO}-\mathrm{HL}‐\mathrm{in}‐\mathrm{LNO}-\mathrm{LL}}=\underset{I\in \mathrm{HL}}{\sum }\delta E_{I}^{\mathrm{LNO}-\mathrm{HL}}+\underset{J\in \mathrm{LL}}{\sum }\delta E_{J}^{\mathrm{LNO}-\mathrm{LL}}$$

Here, $\delta E_{I}^{\mathrm{LNO}-\mathrm{HL}}$ and $\delta E_{J}^{\mathrm{LNO}-\mathrm{LL}}$ collect the correlation energy contributions of LMOs *I* assigned to the active (HL) and LMOs *J* assigned to the environment (LL) layers, respectively. The selection of all LNO methods is supported for both HL and LL methods. Moreover, the LNO convergence accuracy can also be increased for the HL systems, e.g., from Normal to Tight LNO settings. Particularly powerful combinations include LNO-CCSD­(T)-in-LMP2 and more relevantly for the present work, e.g., LNO-CCSDT­(Q)-in-LNO-CCSD­(T). For completeness, let us note that the two-level LNO-based correlation embedding scheme can be further embedded into a third, DFT layer and/or a fourth, classical molecular mechanics (MM) layer.^52^ For example, the enzyme reaction example reported here employs a three-layer LNO-CCSDT­(Q)-in-LNO-CCSD­(T)/MM embedding, as detailed below.

## Computational Details

The calculations were performed with the Mrcc program suite.^46–48^ LNO-CC methods^37,39–42,45^ of Mrcc were assessed against canonical CCSD­(T),^2,3^ CCSDT,^74^ CCSDT­(Q),^5,6^ and CCSDTQ.^75^ The atomic (H, B–F, Al–Cl) open-shell calculations were performed using the 2024 version of Mrcc with the default localcc=2024, as 2024 is the first version of Mrcc supporting open-shell local correlation calculations for CC methods.^42,43^ The Normal, Tight, and veryTight threshold combinations for LNO-CC, set via the lcorthr keyword, were employed as defined in Mrcc. We employed the localcc=2021 setting in Mrcc version 2023 for the closed-shell species in W4-17. It should be noted that, while the latest localcc=2024 is not identical in all aspects, for such small closed-shell systems the results and conclusions are unaffected. In general, the latest localcc=2024 LNO-CC settings are recommended for both closed-shell and open-shell applications.

Correlation-consistent cc-pV*n*Z (*n* = D, T, Q) basis sets by Dunning^76,77^ were used. In some cases, we truncated their high angular momentum functions to match the settings of the reference computations. For example, for cc-pVDZ­(d,s), *p* functions on hydrogen atoms were excluded; for cc-pVTZ­(f,p), *d* functions on hydrogen atoms were omitted; and for cc-pVQZ­(f,d), *g* and *f* functions were removed from non-hydrogen and hydrogen atoms, respectively. We will refer to these basis sets as VDZ­(d,s), VTZ­(f,p), and VQZ­(f,d) throughout this paper.

Molecular geometries for the W4-17 thermochemical benchmark set^11^ were taken from the Supporting Information of ref 11 without modification to evaluate TAE and BSE properties. TAE, the energy required to dissociate a molecule into its atoms, reflects bond strengths and molecular stability, and is a rigorous test for the energetics of small molecules. BSE is defined here as the reaction that separates a molecule into the most stable hydrides of its constituent atoms (e.g., H_2_, BH_3_, CH_4_, NH_3_, H_2_O, HF, AlH_3_, SiH_4_, PH_3_, H_2_S, and HCl). For example, the BSE for CCl_4_ corresponds to the reaction CCl_4_ + 4 H_2_ → CH_4_ + 4 HCl. Our benchmarking of LNO-CC methods is made against the corresponding canonical CC methods. From this set of 160 closed-shell species, we performed LNO-CCSDT and LNO-CCSDT­(Q) calculations. With the VDZ­(d,s) basis set, we obtained CC energies for all species except for C_2_Cl_6_. This was extended to 142 species with the VTZ­(f,p) basis set. Finally, LNO-CCSDT/VQZ­(f,d) calculations were performed for the closed shell molecules in the “W4.3″ subset of the W4-17 data set, for which the W4.3 energies were reported in previous work (see ref 78 and references therein).

In addition, we considered the 1.0 *r*_*e*_ slice of the S66 × 8 data set^58^ of noncovalent interactions, where the canonical CCSDT­(Q) results of refs 79 and 80 are used as reference. Due to computational cost, canonical CCSDT­(Q) was reported not to be feasible for all dimers.^79^ Similarly, because of long wall times, Tight LNO-CCSDT­(Q) computations were not performed for dimers 19, 31, 32, 33, 38, 44, 50, 54, 58, 66. We carried out conventional (i.e., not density fitting) HF, while for the correlation part, a relatively large, cc-pVQZ-RI auxiliary basis set^81^ was applied to reduce the effect of density fitting.

The BH28 structures were taken from ref 69, including the reaction barrier heights of pericyclic (BHPERI), bipolar cycloaddition (CADBH), cycloreversion (CRBH), multiple proton exchange (PXBH), and other reactions (BHDIV). We performed conventional HF and used the cc-pVQZ-RI auxiliary basis set for the LNO-CC correlation energies. Following the CCSDT­(Q) computations of ref 69, we also employed the VDZ­(p,p) and VDZ­(d,p) AO basis sets. To accelerate the computations, we exploited the spatial symmetry of the BH28 structures in the local calculations by setting the localcorrsymm=on option.

The semibullvalene reactant and TS complex structures of the Cope rearrangement reaction were taken from ref 82, and the canonical reference barrier heights were computed with Mrcc. Following the choice of ref 82, we utilized the VDZ­(p,p) AO basis set, i.e., the set of d functions removed from heavy atoms. This was combined with the cc-pVDZ-RI-JK HF and cc-pVDZ-RI correlation auxiliary bases for both the canonical and the local calculations.^81,83^

The structures and MM point charges of the enzyme reaction are adapted from ref 53 (see detailed description of the enzyme reaction below). For consistency with the above basis truncations, we removed the d shell from the def2-SVP AO basis set,^84^ which we denote as def2-SVP­(p,p), with def2-QZVPP-RI-JK HF and def2-SVP-RI correlation auxiliary basis sets.^81,83^

Let us briefly note that the VDZ­(p,p) basis set choice is motivated by consistency with the literature, and it is suitable for the present purposes of testing the accuracy of LNO approximations. For production calculations, we expect the full VDZ or the more consistently truncated VDZ­(p,s) options to achieve better accuracy over cost performance.

## Results and Discussion

### Fully Connected Quadruples Substitutions

Table 1 presents error statistics for the contributions of the fully connected quadruples beyond (Q), denoted *T̂*_4_ – (Q), to BSEs and TAEs for two dozen closed-shell species in the W4.3 data set. These terms are calculated as the difference between LNO-CCSDTQ and LNO-CCSDT­(Q) and are compared to the canonical CCSDTQ–CCSDT­(Q) contributions. (See also Table S1 in the Supporting Information for individual contributions.)

**Table 1:** RMSD and Mean Signed Deviation (MSD) (in kcal·mol^–1^) for LNO-[*T̂*_4_ – (Q)] Contributions to Bond Separation Energies (BSE) and Total Atomization Energies (TAE) against Canonical *T̂*_4_ – (Q) for Closed-Shell Species in the W4.3 Data set

	BSE		TAE^a^	
Threshold	RMSD	MSD	RMSD	MSD
VDZ(d,s)				
Total neglect	0.290	0.128	0.304	0.145
Normal	0.002	0.001	0.003	0.000
Tight	0.001	0.000	0.004	0.000
vTight	0.001	0.000	0.004	0.000
VTZ(f,p)				
Total neglect	0.306	0.129	0.315	0.138
Normal	0.008	0.006	0.006	0.003
Tight	0.002	0.001	0.002	–0.001
vTight	0.001	0.000	0.002	–0.001

a An unrestricted HF (UHF) wave function is used in the atomic calculations for TAEs. No noticeable change occurs in statistics when we switch from UHF to restricted open shell HF-(ROHF-)­based [*T̂*_4_ – (Q)] atomic energies.

Neglecting the [*T̂*_4_ – (Q)] contributions from BSEs results in a substantial root-mean-square deviation (RMSD) of 0.290 and 0.306 kcal·mol^–1^ for VDZ­(d,s) and VTZ­(f,p), respectively. However, for the small set at hand, LNO-CCSDTQ with Normal threshold achieves practically canonical accuracy for both basis sets and all thresholds, with the largest RMSDs found just 0.008 kcal·mol^–1^ for VTZ­(f,p). For completeness, using Tight or vTight thresholds eliminates these errors entirely, while their use does not appear to be necessary for [*T̂*_4_ – (Q)] contributions. For TAEs, the picture is very similar. Thus, LNO-CCSDTQ with a double-ζ basis set and Normal LNO threshold reliably reproduces the [*T̂*_4_ – (Q)] part of canonical CCSDTQ.

While we focus on closed-shell species, the calculation of TAEs raises the question of whether a UHF or ROHF reference is more appropriate. From the perspective of using UHF-based canonical CCSDTQ references, UHF could be considered a slightly better match for LNO-CC. However, since the UHF reference is always projected to a QRO reference before LNO-CC, the QRO- and ROHF-based LNO-CC results are usually very close, when there is little spin symmetry breaking in the UHF solution. This is what we find, e.g., using the Normal threshold, as the MSDs are near −0.02 mE_h_ for the first and second row atoms. Consequently, this conclusion holds irrespectively of the LNO accuracy setting in LNO-CCSDT­(Q) and LNO-CCSDTQ.

For TAEs, the RMSD between LNO-[*T̂*_4_ – (Q)] and canonical terms is 0.009 kcal·mol^–1^ for ROHF and just 0.006 kcal·mol^–1^ for UHF-based CC results. The RMSD values drop to nearly zero for Tight or vTight thresholds, indicating that Normal settings are sufficient here. Given that the choice of reference has no material effect here, either UHF or ROHF could be a viable option for high-level LNO-CC calculations. This finding is similar to what is observed up to LNO-CCSD­(T) and can be explained by the fact that the difference between the QRO and ROHF references is missing some orbital relaxation that would equate the QRO orbitals exactly with the variational ROHF solution. The majority of this relaxation is recovered by the singles orbital rotation part of CCSD. Thus, one can often prioritize practical considerations in the reference choice, for example, that UHF/UKS optimizations can be simpler to converge than corresponding ROHF/restricted open-shell KS.

### Higher-Order Connected Triples and Perturbative Quadruples

Table 2 shows the error statistics for the LNO-based [*T̂*_3_ – (T)], (Q), and [(Q) – (T)] contributions to BSEs against the corresponding canonical corrections for W4-17. Table 3 shows similar error statistics for TAEs. The errors differ since the BSE involves a partial breakdown of the molecule into smaller fragments rather than separate atoms, yielding smaller absolute values than TAEs.

**Table 2:** RMSD and MSD (in kcal·mol^–1^) for LNO-[*T̂*_3_ – (T)], LNO-(Q), and LNO-[(Q) – (T)] Contributions to BSEs with Respect to the Canonical Terms for Species in W4-17

	LNO-[*T̂*_3_ – (T)]		LNO-(Q)		LNO-[(Q) – (T)]	
Threshold	RMSD	MSD	RMSD	MSD	RMSD	MSD
VDZ(d,s)						
Total neglect	0.386	0.207	0.806	–0.530	0.616	–0.322
Normal	0.071	0.040	0.041	–0.018	0.040	0.022
Tight	0.032	0.012	0.017	–0.004	0.018	0.009
vTight	0.014	0.005	0.008^b^	–0.003^b^	0.008^b^	0.002^b^
VTZ(f,p)						
Total neglect	0.721	0.515	1.051	–0.724	0.586	–0.218
Normal	0.115	0.083	0.068	–0.050	0.061	0.033
Tight	0.050	0.033	0.031	–0.021	0.026	0.012
vTight	0.021	0.010	0.014	–0.009	0.013	0.002
VQZ(f,d)^a^						
Total neglect	0.576	0.291	n/a	n/a	n/a	n/a
Normal	0.025	–0.008	n/a	n/a	n/a	n/a
Tight	0.014	–0.009	n/a	n/a	n/a	n/a
vTight	0.006	–0.004	n/a	n/a	n/a	n/a

a 26 closed-shell species from the W4.3 subset are considered.

b Excluded species: N_2_O_4_ and C_2_Cl_4_. Including these two species yields an RMSD of 0.035 kcal/mol and MSD of −0.006 kcal/mol for LNO-(Q). For LNO-[(Q) – (T)], we obtain an RMSD of 0.032 kcal/mol and an MSD of −0.001 kcal/mol.

**Table 3:** RMSD and MSD (in kcal·mol^–1^) for LNO-[*T̂*_3_ – (T)], LNO-(Q), and LNO-[(Q) – (T)] Contributions to TAEs with Respect to the Canonical Terms for Species in W4-17^a^

	LNO-[*T̂*_3_ – (T)]		LNO-(Q)		LNO-[(Q) – (T)]	
Threshold	RMSD	MSD	RMSD	MSD	RMSD	MSD
VDZ(d,s)						
Total neglect	0.315	0.119	1.236	–0.980	1.102	–0.860
Normal	0.121	0.018	0.043	–0.023	0.127	–0.005
Tight	0.060	0.032	0.024	–0.012	0.056	0.020
vTight	0.072	0.045	0.014^c^	–0.007^c^	0.063^c^	0.038^c^
VTZ(f,p)						
Total neglect	0.906	0.727	1.362	–1.052	0.666	–0.340
Normal	0.102	0.070	0.079	–0.062	0.044	0.008
Tight	0.037	0.016	0.036	–0.026	0.021	–0.011
vTight	0.019	–0.006	0.016	–0.011	0.024	–0.017
VQZ(f,d)^b^						
Total neglect	0.717	0.479	n/a	n/a	n/a	n/a
Normal	0.028	0.011	n/a	n/a	n/a	n/a
Tight	0.011	–0.008	n/a	n/a	n/a	n/a
vTight	0.014	–0.010	n/a	n/a	n/a	n/a

a The UHF reference is used in all atomic energies.

b 26 closed-shell species from the W4.3 subset are considered.

c Excluded species: N_2_O_4_ and C_2_Cl_4_. Including these two species yields an RMSD of 0.038 kcal/mol and MSD of −0.011 kcal/mol for LNO-(Q). For LNO-[(Q) – (T)], we obtain an RMSD of 0.071 kcal/mol and an MSD of 0.034 kcal/mol.

For a reference point, let us establish first the total size of these contributions, denoted in the tables as “total neglect”. With the smaller basis sets, the neglect of [*T̂*_3_ – (T)] leaves a 0.386 kcal·mol^–1^ RMSD for VDZ­(d,s), a sizable 0.721 kcal·mol^–1^ for VTZ­(f,p), which reduces to 0.576 kcal·mol^–1^ for VQZ­(f,d). The (Q) contributions are somewhat larger, and only minor cancellation is observed when adding these into the [(Q) – (T)] contribution. Compared to that, Normal LNO settings already recover 85–95% of this effect. Namely, we find 0.041–0.115 kcal·mol^–1^ (0.1–0.5 kJ·mol^–1^) RMSDs across all terms and all basis sets, with an excellent, sub-0.25 kJ·mol^–1^ performance for the [(Q) – (T)] contribution. If needed, Tight LNO settings can bring the RMSDs further down by more than a factor of 2, which reads for [*T̂*_3_ – (T)] as just 0.032 kcal·mol^–1^ for VDZ­(d,s), 0.050 kcal·mol^–1^ for VTZ­(f,p), and a mere 0.014 kcal·mol^–1^ for VQZ­(f,p). The errors are even more controlled for LNO-(Q) and LNO-[(Q) – (T)], with RMSD values generally being smaller than those for triples, which is expected, given the more rapid convergence of higher-order excitations in the CC expansion. For example, the 0.018–0.026 kcal·mol^–1^ for Tight LNO-[(Q) – (T)] are already better than needed for practical applications. For completeness, vTight LNO settings bring another more than 2× improvement over Tight LNO for the BSEs across the board, reaching the 0.1 kcal·mol^–1^ accuracy mark.

Turning to the TAEs in Table 3, the LNO performance is highly analogous, with the notable difference of the somewhat larger overall contributions across [*T̂*_3_ – (T)], (Q), and [(Q) – (T)]. However, both relative and absolute LNO errors are well below the limit of practical use, especially considering the Normal and Tight LNO TAE RMSDs of 0.044 and 0.021 kcal·mol^–1^ obtained for [(Q) – (T)]/VTZ­(f,p). One exception to note is the vTight LNO behavior with the smallest, VDZ­(d,s) basis set, where vTight does not uniformly improve the RMSD over Tight LNO. The culprits are N_2_O_4_ and C_2_Cl_4_; upon their exclusion, the RMSD decreases, e.g., for BSE (Q)/VDZ­(d,s), from 0.035 to 0.008 kcal·mol^–1^ (Table 2). All in all, the LNO accuracy improves from [*T̂*_3_ – (T)] through (Q) to [(Q) – (T)], suggesting that less tight cutoffs are sufficient for higher CC orders, in accord also with the DLPNO–CCSDT­(Q) study by Jiang et al.^85^

Table 4 shows the statistical analysis of the basis set convergence for LNO-based [*T̂*_3_ – (T)], (Q), and [(Q) – (T)] against the canonical CC results with the VQZ­(f,d) basis set. Let us note that the choice of UHF or ROHF reference for the atomic energies does not affect the accuracy for either LNO- or canonical CC; thus, ROHF-based results are not discussed further. Starting with the basis set incompleteness error of the canonical results, unsurprisingly the VDZ­(d,s) basis set is insufficient, yielding 0.163–0.421 kcal·mol^–1^ RMSDs for BSE and about twice as large RMSDs for TAEs. Compared to that, VTZ­(f,p) basis set errors shrink to 0.035–0.050 kcal·mol^–1^ for BSEs and about 3 times that high for TAEs.

**Table 4:** RMSD Values (in kcal·mol^–1^) for LNO-CCSDT and LNO-CCSDT­(Q) Correlation Energy Contributions against Canonical CCSDT and CCSDT­(Q) with VQZ­(f,d) Basis Set as Reference, Respectively, for Energetics in the W4-17 Data Set

	BSE for LNO-CC			TAE for LNO-CC (UHF)			TAE for LNO-CC (ROHF)		
Threshold	*T̂*_3_ – (T)	(Q)	(Q) – (T)	*T̂*_3_ – (T)	(Q)	(Q) – (T)	*T̂*_3_ – (T)	(Q)	(Q) – (T)
Normal/VDZ(d,s)	0.471	0.318	0.185	0.896	0.311	0.628	0.885	0.307	0.617
Tight/VDZ(d,s)	0.436	0.303	0.169	0.893	0.301	0.646	0.881	0.297	0.634
vTight/VDZ(d,s)	0.428	0.288^a^	0.162^a^	0.902	0.288^a^	0.646^a^	0.890	0.284^a^	0.634^a^
can/VDZ(d,s)	0.421	0.299	0.163	0.859	0.294	0.630	0.838	0.288	0.612
Normal/VTZ(f,p)	0.149	0.098	0.075	0.249	0.154	0.098	0.237	0.154	0.090
Tight/VTZ(f,p)	0.085	0.062	0.044	0.183	0.111	0.072	0.171	0.111	0.063
vTight/VTZ(f,p)	0.058	0.047	0.034	0.155	0.093	0.064	0.144	0.093	0.056
can/VTZ(f,p)	0.050	0.042	0.035	0.174	0.087	0.084	0.173	0.088	0.082

a Excluded species: N_2_O_4_ and C_2_Cl_4_. Including these two species yields LNO-(Q)’s RMSD of 0.313, 0.308, and 0.304 kcal/mol for BSE, TAE­(UHF) and TAE­(ROHF), respectively, and similarly LNO-[(Q) – (T)]’s RMSD of 0.164, 0.656, and 0.644 kcal/mol, respectively.

These canonical VDZ­(d,s) and VTZ­(f,p) errors provide clear performance targets for the LNO approximations. With the VDZ­(d,s) basis set, the basis set error dominates both BSEs and TAEs, even Normal LNO yielding practically canonical quality performance. Considering VTZ­(f,p), Normal LNO adds some uncertainty on top of the basis set error, especially for the worst case of [*T̂*_3_ – (T)] contributions to BSEs, where an additional 0.1 kcal·mol^–1^ deviation emerges. However, for the [(Q) – (T)]/VTZ­(f,p) term, also for BSEs and especially for TAEs, Normal LNO errors are below the residual basis set error and thus provide a quality almost as useful as canonical results, well below 0.1 kcal·mol^–1^ accuracy with respect to canonical VQZ­(f,d). If needed, LNO errors for VTZ­(f,p) can be made negligible compared to the basis set error via Tight settings, e.g., yielding only 0.01 kcal·mol^–1^ LNO errors for [(Q) – (T)]/VTZ­(f,p). Here, however, a more balanced Normal LNO VTZ­(f,p) has a favorable accuracy over the cost profile, and the next balanced combination appears to be Tight LNO VQZ­(f,d), or rather a composite approach using tighter settings with VTZ­(f,p) combined with Normal LNO level basis set correction from VTZ­(f,p) to VQZ­(f,d).

### Convergence for Perturbative Triples

While comprehensive accuracy assessment of LNO performance is available up to LNO-CCSD­(T) (see review in ref 45.), due to its role, e.g., in [(Q) – (T)] corrections, we briefly inspect the (T) contribution by itself. The statistical analysis for the (T) contribution (taken as LNO-CCSD­(T) – LNO-CCSD) to BSEs for the W4-17 data set is shown in Table 5. Since the (T) term is substantially larger, its LNO errors are also larger than for higher-order CC terms. As detailed in previous works,^45^ the LNO threshold combinations are optimized for LNO-CCSD­(T) in combination with sufficiently large, that is, triple- and quadruple-ζ basis sets. In accord with previous assessments, the LNO errors for (T) are somewhat higher for the small VDZ­(d,s) basis, considering this the 0.304 kcal·mol^–1^ RMSD for (T)/VDZ­(d,s) is satisfactory with Normal LNO settings. Compared to that, we find with VTZ­(f,p) and VQZ­(f,d) 2–4 times better Normal LNO accuracy, at the 0.15 kcal·mol^–1^ scale. Clearly, the (T)/VDZ­(d,s) combination has such high basis set incompleteness error that it is not useful by itself, whereas we see that Normal LNO works well when considering the post-(T) effects. Shifting our focus to (T)/VTZ­(f,p) and (T)/VQZ­(f,d), Tight settings bring almost a factor of 2 improvement. Upon switching to vTight, the RMSD plunges to 0.023–0.050 kcal·mol^–1^ for all three basis sets.

**Table 5:** RMSD and MSD (in kcal·mol^–1^) for LNO-(T) Contribution to BSEs with Respect to Canonical (T) for Species in the W4-17 Data Set. Numbers in Parentheses Label Changes When Switching from the Default ccprog=ccsd to ccprog=mrcc

Threshold	VDZ(d,s)	VTZ(f,p)	VQZ(f,d)
RMSD			
Normal	0.304 (+0.263)	0.145 (−0.005)	0.166 (+0.046)
Tight	0.284 (+0.279)	0.077 (+0.005)	0.085 (unch)
vTight	0.023 (unch)	0.046 (unch)	0.050 (+0.002)
MSD			
Normal	–0.217 (−0.238)	–0.028 (−0.001)	–0.120 (+0.070)
Tight	–0.239 (−0.238)	–0.041 (−0.007)	–0.043 (+0.001)
vTight	0.003 (−0.002)	–0.017 (unch)	–0.019 (−0.003)

Let us briefly inspect the effect of using ccprog=mrcc, with results given in parentheses in Table 5. For (T)/VDZ­(d,s), we can see a notable change that is connected to the relatively large effect of the NAF approximation for the small VDZ­(d,s) basis set. Namely, a much smaller number of auxiliary functions are retained for the small VDZ­(d,s) AO basis. Compared to that, the (T)/VTZ­(f,p) and (T)/VQZ­(f,d) results are practically the same with the ccprog=ccsd and ccprog=mrcc approaches. So let us highlight again that the use of ccprog=mrcc is not recommended for (T) by itself, just when these NAF errors are canceled when forming [*T̂*_3_ – (T)] or [(Q) – (T)] corrections, as demonstrated above.

### Effect of BSSE on LNO-Based Post-CCSD­(T) Corrections for Thermochemistry

In recent work,^80^ we considered the BSSE effects on post-CCSD­(T) corrections for a subset of diatomic and triatomic species from the W4-17 benchmark, in the context of canonical CC theory. We now pose the following question: Are the post-LNO-CCSD­(T) corrections affected by BSSE in the same qualitative and quantitative manner as their canonical post-CCSD­(T) counterparts?

Table 6 shows the results for various post-(T) contributions to the counterpoise-corrected TAEs against the canonical reference. First, the LNO-CC methods generally underestimate the BSSE-corrected canonical contributions, regardless of the chosen basis set and accuracy threshold. Second, compared to [*T̂*_3_ – (T)], for the (Q) correction, there is a milder dependence on the LNO settings. Already, the Normal threshold reproduces the canonical (Q) contribution to within a few hundredths of a kcal·mol^–1^, consistent with the very rapid basis set convergence of both canonical and local quadruples.

**Table 6:** RMSD (kcal·mol^–1^) of LNO Approximation for Counterpoise-Corrected Post-CCSD­(T) Correlation Energy Components to the TAEs for a Set of Diatomic and Triatomic Closed-Shell Species from the W4-17 Benchmark Data Set

Contribution	Threshold	VDZ(d,s)	VTZ(f,p)	VQZ(f,d)
*T̂*_3_ – (T)	Normal	0.153	0.196	0.091
*T̂*_3_ – (T)	Tight	0.131	0.178	0.079
*T̂*_3_ – (T)	vTight	0.110	0.129	0.060
(Q)	Normal	0.014	0.040	0.059
(Q)	Tight	0.014	0.027	0.029
(Q)	vTight	0.015	0.021	0.022
(Q) – (T)	Normal	0.150	0.203	0.123
(Q) – (T)	Tight	0.131	0.188	0.083
(Q) – (T)	vTight	0.110	0.121	0.064

Overall, the picture for LNO-based post-(T) BSSE effects is very similar to that of canonical post-(T) corrections in thermochemistry. BSSE on the LNO-(Q) contributions is negligible for the studied diatomics and triatomics, much like the negligible BSSE found for canonical connected quadruples, even with relatively modest basis sets, in previous work by the Martin group.^80^ BSSE on the LNO-[*T̂*_3_ – (T)] term is larger, but still modest in absolute terms and further reduced as we move to tighter LNO thresholds and larger basis sets, again mirroring the canonical behavior, where BSSE rapidly diminishes with basis set. Finally, the BSSE-corrected contributions beyond LNO-CCSD­(T) are essentially insensitive to the choice of UHF versus ROHF reference. Within the statistical noise of the present data, the RMSDs for the two references are indistinguishable.

## Accuracy for Barrier Heights

We continue with the barrier height energy analysis of the BH28 test set in Table 7 (see also Table S2 in the Supporting Information). First, we look at the post-CCSD­(T) contributions as a whole (rows “Total neglect”) against the canonical CCSDT­(Q) for both VDZ­(p,p) and VDZ­(d,p) basis sets, motivated by ref 69. Clearly, the effect of removing the polarization capability of d functions from VDZ­(d,p) (i.e., from conventional cc-pVDZ) results in significant underestimation of the post-(T) for all three types of post-CCSD­(T) corrections. For example, the RMSD of the total (Q) – (T) correction computed with VDZ­(d,p) is 50% higher than with VDZ­(p,p). Next, let us note the increase of the RMSDs in the direction of *T̂*_3_ – (T), (Q), and (Q) – *T̂*_3_ with both basis sets, cf. 0.483, 0.597, and 0.647 kcal·mol^–1^, respectively, for VDZ­(d,p). Let us also consider, in comparison to the RMSDs, the similarly sized MSDs for (Q) and the much smaller MSDs for *T̂*_3_ – (T). Thus, (Q) appears to stabilize consistently, while the effect of *T̂*_3_ – (T) can fairly equally stabilize and destabilize the TS. Consequently, their combination into (Q) – (T) does not lead to cancellation; instead, (Q) – (T) is slightly higher than both *T̂*_3_ – (T) and (Q), at least for this BH28 barrier data set. At this point, it is important to highlight the relatively challenging nature of the BH28 barrier compilation. As it collects pericyclic, cycloaddition, cycloreversion, and multiple simultaneous proton exchange barriers, the average number of forming and breaking bonds is relatively high (about 5–6) compared to the case of more common reactions. Since the post-CCSD­(T) terms are consequently higher than usual, such reactions are thus important targets of post-CCSD­(T) models.

**Table 7:** Statistics of the Post-CCSD­(T) Barrier Height Energy Correction Sizes and Local Approximation Errors for the BH28 Test Set with Respect to the Canonical CCSDT­(Q) Reference.^a^

	*T̂*_3_ – (T)		(Q)		(Q) – (T)	
	RMSD	MSD	RMSD	MSD	RMSD	MSD
VDZ(p,p)						
Total neglect	0.282	–0.040	0.365	0.312	0.432	0.272
Loose	0.129	–0.110	0.091	0.075	0.064	–0.035
Normal	0.037	–0.017	0.031	0.025	0.024	0.008
Tight	0.014	0.000	0.011	0.006	0.013	0.006
VDZ(d,p)^b^						
Total neglect	0.483	–0.099	0.597	0.519	0.647	0.420
Loose	0.121	–0.095	0.128	0.114	0.092	0.018
Normal	0.053	–0.026	0.062	0.054	0.048	0.028

a Energy Unit: kcal·mol^–1^

b BHPERI subset was set aside from VDZ­(d,p) calculations due to the higher wall-clock time requirement.

Turning to the accuracy of LNO variants in Table 7, we find a consistent LNO performance for both VDZ­(p,p) and VDZ­(d,p), for all three post-CCSD­(T) correction types. For example, the (Q) – (T) RMSDs with the 2 basis sets are 14.8% and 14.3% with Loose and 5.6% and 7.4% with Normal thresholds. Tightening the LNO thresholds yields the expected systematic improvement. For example, for VDZ­(d,p), the relative RMSDs compared to the total effect are 14–25% with Loose, 7–10% with Normal, and, for VDZ­(p,p), 3–5% with Tight LNO settings for all three corrections. When inspecting also the MSDs in comparison to the RMSDs, the LNO errors are very systematically negative for *T̂*_3_ – (T) and positive for (Q), resulting in some error compensation to the total (Q) – (T) correction. All in all, the 0.064–0.092 kcal·mol^–1^ Loose LNO and especially the twice as good 0.024–0.048 kcal·mol^–1^ Normal LNO RMSDs bring in 93–95% of the (Q) – (T) effect and are thus highly suitable for practical use in thermochemistry, even for challenging barriers like those in BH28.

To push the barrier tests further, we consider one of the largest systems where canonical CCSDT­(Q) is available, namely the Cope rearrangement of semibullvalene (Figure 1). The system size increases to 16 atoms and 12 bonds, directly affected by the bond rearrangement in this TS. The canonical VDZ­(p,p) reference in Table 8 shows −0.139, 0.268, and 0.129 kcal·mol^–1^ for the *T̂*_3_ – (T), (Q), and (Q) – (T) corrections. These are, compared to the BH28 MSDs in the same basis set, slightly higher for *T̂*_3_ and lower for (Q). The LNO approximations again improve consistently, that is, for *T̂*_3_ – (T) and (Q), the LNO error decreases from 17–25% to 5–9%, when switching from Loose to Normal settings. Since also the opposite-signed canonical *T̂*_3_ – (T) and (Q) show some cancellation, the LNO (Q) – (T) results also benefit from this, yielding a highly pleasing 0.013–0.026 kcal·mol^–1^ (Q) – (T) accuracy with both Loose and Normal LNO settings.

**Table 8:** Post-CCSD­(T) Barrier Height Energy Correction Size and Local Approximation Errors (in kcal·mol^–1^) of the Cope Rearrangement of Semibullvalene Reaction with Respect to the Canonical Results, Using VDZ­(p,p)

	*T̂*_3_ – (T)	(Q)	(Q) – (T)
Total neglect	–0.139	0.268	0.129
Loose	–0.035	0.048	0.013
Normal	0.013	0.013	0.026

**Figure 1:**
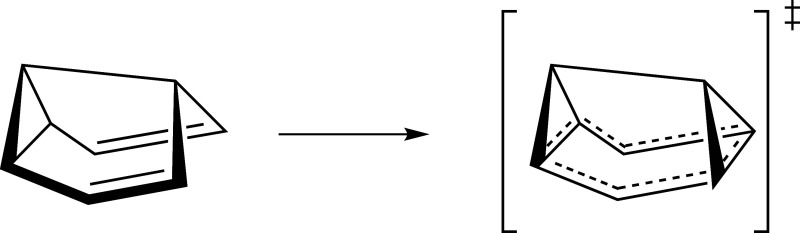
Reaction pathway yielding the transition state complex of semibullvalene Cope rearrangement.

### LNO-Based Post-CCSD­(T) for Noncovalent Interactions

It is also of high interest to examine the post-(T) contributions—specifically, the *T̂*_3_ – (T) and (Q) terms—for noncovalent interactions. Building on recent work from the Martin group,^79^ we utilize CCSDT­(Q)/VDZ­(d,s) references obtained for the 1.00*r*_*e*_ slice of the S66 × 8 data set to test new LNO-CCSDT­(Q) results. The post-CCSD­(T) correction sizes and LNO error statistics are given in Table 9 for the conventional hydrogen bonds, dispersion, and mixed subsets too (see also Table S3 in the Supporting Information for the individual interaction energy contributions).

**Table 9:** Post-CCSD­(T) Interaction Energy Correction Size and Local Approximation Errors (kcal·mol^–1^) for the S66 × 8 (1.00) Test Set with Respect to the Canonical Results, Using the VDZ­(d,s) Basis Set

	*T̂*_3_ – (T)		(Q)		(Q) – (T)	
	RMSD	MSD	RMSD	MSD	RMSD	MSD
Hydrogen bonds						
Total neglect	0.020	–0.014	0.020	0.017	0.018	0.003
Loose	0.044	–0.033	0.029	0.025	0.017	–0.008
Normal	0.040	–0.032	0.025	0.019	0.016	–0.013
Tight	0.021	–0.017	0.018	0.013	0.005	–0.004
Dispersion interactions						
Total neglect	0.063	–0.054	0.052	0.050	0.018	–0.005
Loose	0.075	–0.066	0.049	0.046	0.030	–0.020
Normal	0.056	–0.053	0.032	0.031	0.024	–0.022
Tight	0.037	–0.036	0.018	0.017	0.020	–0.019
Mixed interactions						
Total neglect	0.047	–0.040	0.037	0.032	0.011	–0.007
Loose	0.053	–0.047	0.037	0.034	0.018	–0.012
Normal	0.045	–0.039	0.026	0.024	0.019	–0.015
Tight	0.032	–0.027	0.017	0.015	0.016	–0.012
All						
Total neglect	0.040	–0.030	0.034	0.028	0.016	–0.002
Loose	0.054	–0.044	0.036	0.032	0.021	–0.012
Normal	0.045	–0.038	0.027	0.023	0.019	–0.015
Tight	0.026	–0.021	0.017	0.014	0.011	–0.007

Inspecting the size of the canonical post-CCSD­(T) terms first, one finds these interaction energy contributions to be at least an order of magnitude smaller than for the reactions and barriers above. Namely, 0.040, 0.034, and 0.016 kcal·mol^–1^ RMSD is found for the complete data set for the *T̂*_3_ – (T), (Q), and (Q) – (T) terms, respectively. These values are representative of the mixed subset, while the *T̂*_3_ – (T) and (Q) RMSDs for the hydrogen bonding and dispersion subsets are about a factor of 2 smaller and 1.5 times larger, respectively. The larger post-CCSD­(T) contributions to the dispersion subset can be mostly attributed to π orbital interactions. Interestingly, (Q) – (T) corrections are consistently small (0.011–0.018 kcal·mol^–1^) for all three subsets, because of the mostly repulsive *T̂*_3_ – (T) and attractive (Q) terms.

The small size of these post-CCSD­(T) terms also affects the LNO error trends. Specifically, although the error statistics generally improve as the truncation thresholds are tightened (Loose to Normal to Tight), the improvement is not always monotonic and, in terms of relative error, the convergence is slower than in the preceding benchmark cases. Consequently, the post-CCSD­(T) effect sizes and the residual LNO errors become comparable in size. The Loose error statistics can even slightly surpass the canonical post-CCSD­(T) effect for all three kinds of terms. Thus, Loose LNO in the context of noncovalent interactions is useful only for qualitatively assessing the magnitude of the post-CCSD­(T) contribution. Normal LNO settings still exhibit sizable relative deviations, but in terms of the absolute errors recover post-CCSD­(T) interaction components to about 0.05, 0.03, and 0.02 kcal·mol^–1^ accuracy in the *T̂*_3_ – (T), (Q), and (Q) – (T) terms, respectively. This could be already of practical use, providing accuracy at the conservative standard of 0.1 kcal·mol^–1^ often assigned for interaction energy predictions. This accuracy is also sufficient to decide on the need for post-CCSD­(T) corrections in most applications, at least in this size range. Tight LNO settings improve this performance by almost a factor of 2, so their use can be justified when aiming for additional confidence, if allowed by the computational cost.

Regarding the sign of the LNO errors, one also finds a consistent pattern. *T̂*_3_ – (T) MSDs are mostly negative, systematically canceled by mostly positive MSDs for the (Q) terms. The resulting (Q) – (T) contributions thus systematically benefit from LNO error compensation between *T̂*_3_ – (T) and (Q), which holds generally for all three S66 subsets and all three LNO threshold sets. The systematic cancellation also holds for the conventional *T̂*_3_ – (T) and (Q) contributions, making the recovery of the (Q) – (T) results more challenging with local approximations. It is also worthwhile briefly inspecting the largest LNO errors, as the S66 data set is relatively heterogeneous from the perspective of post-CCSD­(T) effects. Namely, negligible (0.001 kcal·mol^–1^ scale) (Q) – (T) results often appear, e.g., for the 13 Peptide···MeOH dimer, while the same correction can grow above 0.05 kcal·mol^–1^, e.g., for the dimers with aromatic interactions. Considering the maximum (Q) – (T) error measures, they are about 2–3 times higher than the RMSDs, consistently across the three S66 subsets as well as for both the canonical post-CCSD­(T) corrections sizes and their LNO errors with all LNO threshold sets.

### Timing Comparison

How much more efficient are LNO-CCSDT and LNO-CCSDT­(Q) compared to their canonical counterparts? To enable direct comparison of LNO and canonical performance, timing data are shown in Table 10 and Figure 2 obtained for small molecules from the W4-11 ^86^ data set, and for various alkanes and some of their dimers in the ADIM6 subset from the GMTKN30 ^87^ database. Point group symmetry was not employed because the canonical and local post-CCSD­(T) codes in Mrcc benefit from markedly different speedups from symmetry, and also because practical molecules targeted by local correlation approaches are rarely symmetric. These jobs ran on same-architecture nodes, equipped with Intel­(R) Xeon­(R) Gold 6240R CPUs operating at 2.40 GHz, 380 GB of memory and 3.6 TB of SSD, with each job running on 16 cores for timing purposes. The Normal accuracy threshold in localized methods was considered in the timing comparison section.

**Figure 2:**
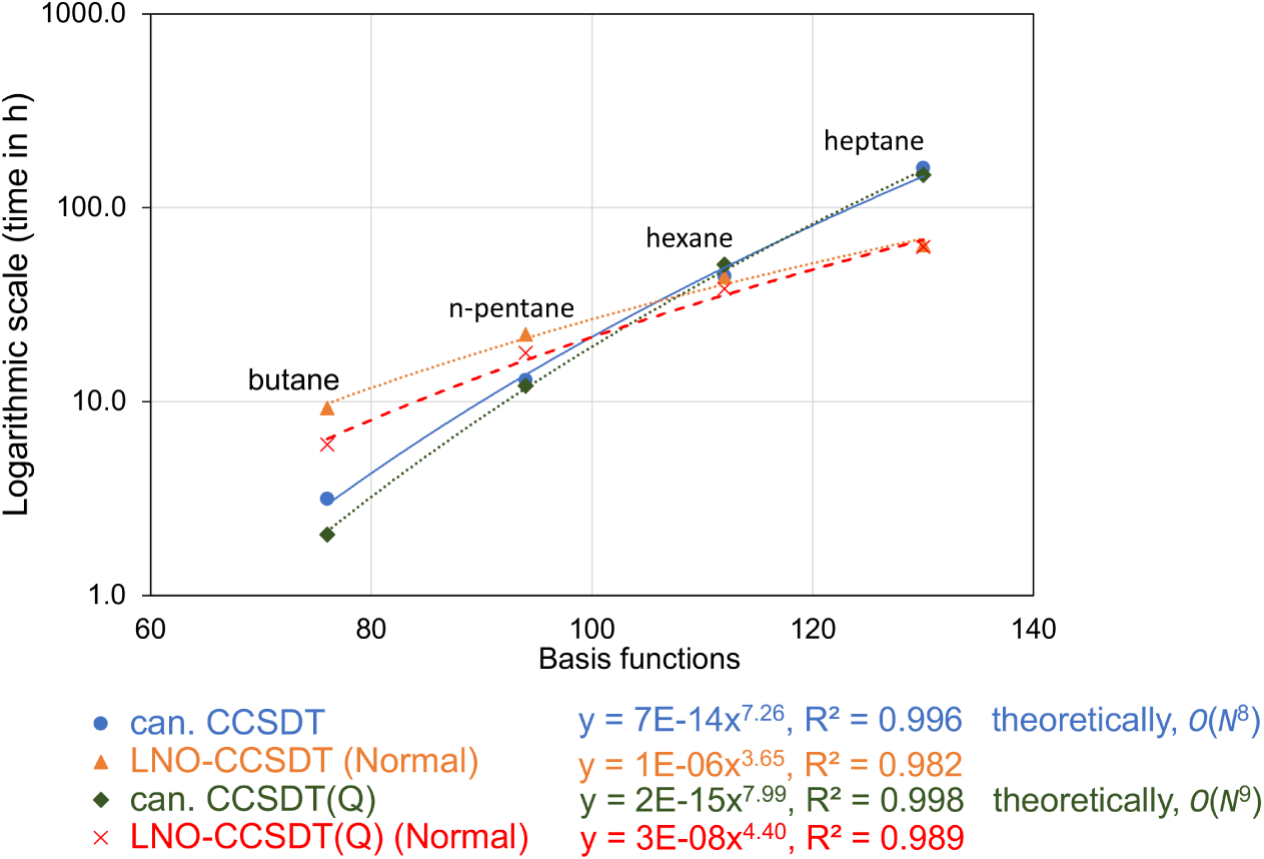
Timing data (in hours) for CCSDT and (Q) corrections in Mrcc for local and canonical CCSDT­(Q) calculations on selected alkanes using the VDZ­(d,s) basis set.

**Table 10:** Timing Data for Selected Species in the W4-17 Data Set

		Time (min)			
Species	Number of basis functions	Can. CCSDT	LNO-CCSDT	Can. (Q)	LNO-(Q)
VDZ(d,s)					
C_2_H_2_	32	1	3	0.5	1
O_3_	42	12	86	2	10.5
NCCN	56	29	140	7	37
BF_3_	56	43	69	15	20
Benzene	96	843	4919	645	3910
(Ethane)_2_	80	277	113	213	38
(Propane)_2_	116	3832	1347	4423	816
VTZ(f,p)					
C_2_H_2_	78	22	33	14	17
O_3_	90	274	1126	132	491
NCCN	120	640	947	676	643
BF_3_	120	1194	701	1518	433
(Ethane)_2_ ^a^	228	3346	1584	19309	1707

a Symmetry was enabled for canonical CCSDT and CCSDT­(Q) calculations.

We start with linear alkane chains, restricted to the small VDZ­(d,s) basis set in order to enable canonical computations through heptane. From ethane to pentane, local correlation methods lag behind their canonical counterparts, while the CPU times are tied for hexane, with major benefits for heptane and beyond. Very good power function fits of the timings data yield scaling exponents of 3.65 for LNO-CCSDT vs 7.26 for canonical CCSDT, and 4.40 for LNO-CCSDT­(Q) vs 7.99 for canonical CCSDT­(Q). Clearly, LNO-CC methods significantly reduce computational cost scaling with system size already for small molecules that are far from the asymptotic linear scaling regime. For heptane, LNO-CCSDT is 2.5 times faster than canonical CCSDT, and similarly, the (Q) correction in LNO-CCSDT­(Q) is obtained 2.3 times more rapidly than in canonical CCSDT­(Q). Another example is the propane dimer. It takes 20.6 h for LNO-CCSDT vs 60 h for canonical CCSDT, and only 13.6 h for LNO-(Q) vs 73.7 h for the canonical (Q) term. The crossover point where localized post-CCSD­(T) methods outperform their canonical counterparts appears to lie between *n*-pentane and *n*-hexane (around 100 basis functions).

A major drawback of this measurement is the use of VDZ­(d,s), as there is practically nothing to compress by the natural orbital approximation in the already incomplete AO basis. Thus, the LNO speedup would be larger with a more practical basis set and also for larger molecules. For such cases, the canonical CCSDT­(Q) scaling would be closer to the theoretical exponent of 9, while the LNO approach would converge toward its asymptotic linear-scaling limit. However, the great expense and steep scaling of canonical CCSDT­(Q) prevents us from going further with such direct comparisons. If we switch to VTZ­(f,p), then for (ethane)_2_, there are 228 basis functions and the wall clock time for LNO-CCSDT­(Q) is 54 h (28.4 h for (Q) and 24.3 h for CCSDT terms). For such a large example, even with the acceleration via point group symmetry, this canonical CCSDT­(Q)/VTZ­(f,p) was not finished after a month. Then, it ran out of disk space with the CCSDT energy nearly converged (<0.00000061 E_h_ from the previous cycle) after 10 iterations. Such disk and memory demand bottlenecks are greatly reduced by the LNO approach. Propane was one of the largest species for which we obtained canonical (Q)/VTZ­(f,p). It required 99.3 h for (Q) vs 25.2 h LNO-(Q), corresponding to a 4-fold speedup. With the smaller VDZ­(d,s), wall times drop to only 0.2 h for canonical (Q) and 1.0 h for LNO-(Q). As explained above, for small systems and small basis sets, especially below 100 orbitals, canonical CCSDT­(Q) could be more advantageous. However, LNO-CCSDT­(Q) excels with both larger species and larger basis sets, requiring much less memory and disk space than parent CCSDT­(Q). Timing data for additional species in the W4-17 data set are shown in Table 10 and data for more species are provided in the Supporting Information. For small species, both for VDZ­(d,s) and VTZ­(f,p), LNO-CCSDT is slower than canonical CCSDT below 80–100 basis functions, then LNO-CCSDT rapidly overtakes it. The crossover point comes a bit earlier for LNO-(Q), which has even larger speedups over canonical CCSDT­(Q) than obtained for CCSDT.

Next, we also assessed the multithreaded (OpenMP) performance of the post-CCSD­(T) methods for calculations on (ethane)_2_ with VDZ­(d,s). All jobs were run exclusively on the same single NUMA node of a system powered by an Intel Xeon Gold 5320 CPU at 2.20 GHz, 768 GB of memory, and 768 GB of SSD. Each job used up to 16 threads. The selected threads were scheduled across the same number of cores, one thread per core, without using hyper-threading. Figure 3 shows timing data as a function of thread number. First, canonical CCSDT peaks at 8 cores, with a speedup of 3.0, and it remains well below the ideal linear speedup of 16. Second, canonical (Q) has an excellent speedup of 14.3 at 16 threads. Third, LNO-CCSDT exhibits moderate scaling, reaching a speedup of 4.3 at 16 threads. Lastly, parallelization is comparably good to its canonical counterpart for the LNO-(Q) correction, yielding a speedup of 13 with 16 cores.

**Figure 3:**
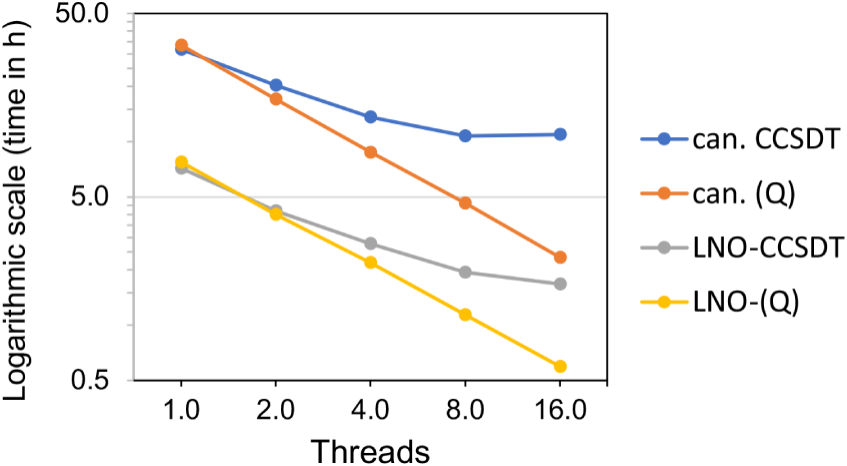
Multithreaded (OpenMP) performance of CCSDT and the (Q) correction from canonical CCSDT­(Q) and LNO-CCSDT­(Q) calculations on (ethane)_2_ using the VDZ­(d,s) basis set. The scale is logarithmic for the *y*-axis.

### Large-Scale LNO-CCSDT­(Q) Application for an Enzyme Reaction

As a proof of principle application, we investigate a methylation reaction catalyzed by the catechol-O-methyltransferase (COMT) enzyme in a QM-in-QM/MM multilevel framework. That is, we employed an LNO-CCSDT­(Q)-in-LNO-CCSD­(T)/MM embedding scheme for the post-(T) contributions to the methylation reaction, utilizing the def2-SVP­(p,p) AO basis set. In this process, shown schematically in Figure 4 and in the protein environment in Figure 5, a methyl group is transferred by the COMT enzyme from the S-adenosyl-l-methionine to the deprotonated catechol amine (denoted as SAM and CAT in Figure 4, respectively). For the QM/MM partition, we follow our previous multilevel work, assigning 571 QM atoms (including 18 link atoms) to the QM region out of the total 3420 protein atoms.^53^ Then, the QM subsystem is partitioned into a higher-level, LNO-CCSDT­(Q) region and a lower-level LNO-CCSD­(T) region. Motivated by which atoms play the most important role in the methylation reaction, we add to the LNO-CCSDT­(Q) layer the Mg ion, the S and O atoms between which the methyl group is transferred, and the 4 atoms of the methyl group itself (red-colored atoms in Figure 4). It is worthwhile noting a useful property of our multilevel LNO methods originating from the definition of our LNO-HL-in-LNO-LL multilayer approach of eq 2. Namely, to obtain the LNO-based (Q) – (T) correction, we only need to evaluate the LNO-CCSDT­(Q) and LNO-CCSD­(T) results for those LMOs that are assigned to the 7 selected atoms in the higher-level subsystem. In practical applications, this LNO-based (Q) – (T) correction should be combined with an LNO-CCSD­(T)/MM computation with a sufficiently large basis set and QM region. Both of the LNO-based (Q) – (T) and larger basis LNO-CCSD­(T) steps are feasible for such large enzyme systems, enabled uniquely by our efficient LNO-CC implementations in Mrcc.^41,45^

**Figure 4:**
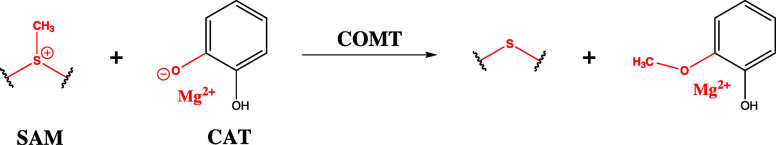
Schematic representation of the methylation reaction catalyzed by the COMT enzyme.

**Figure 5:**
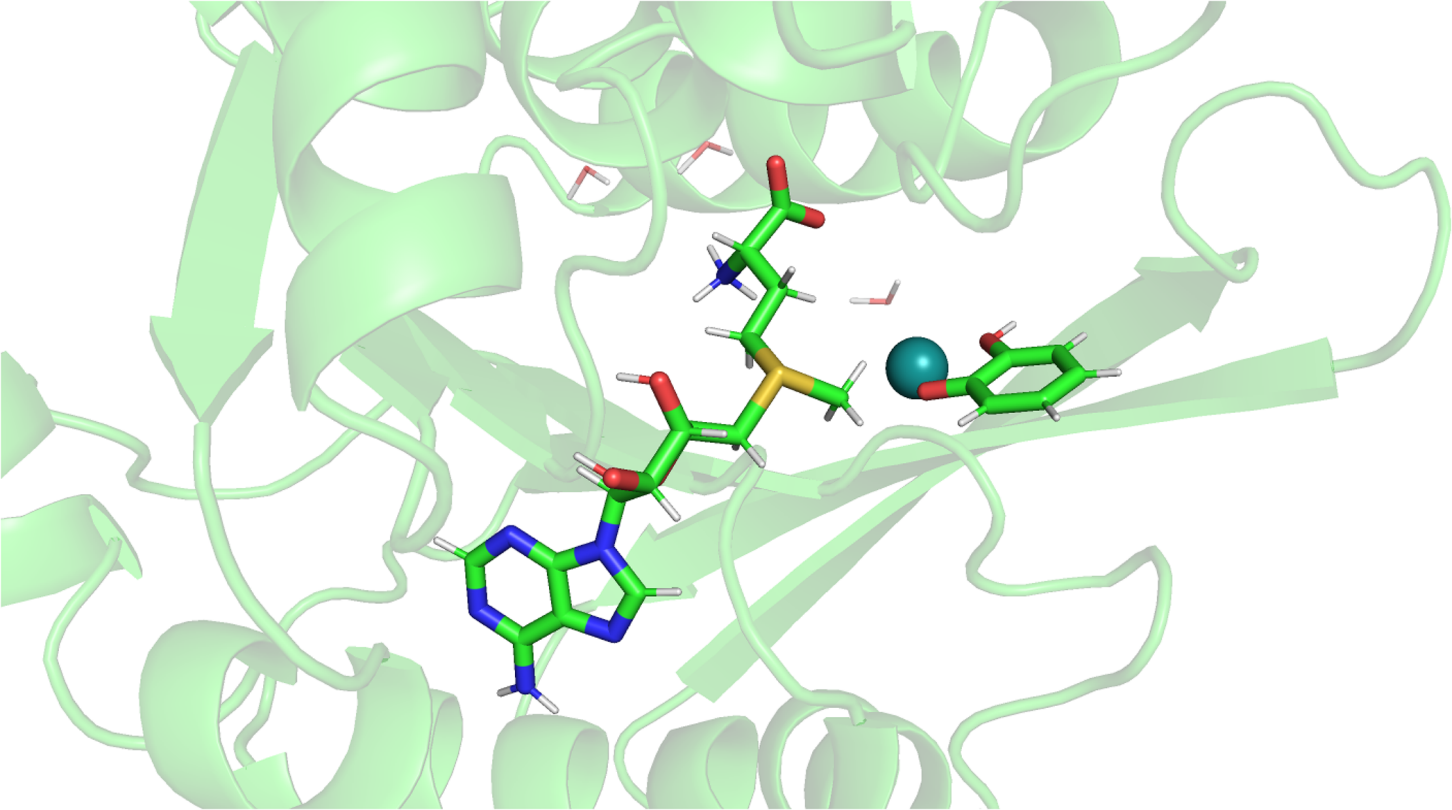
Reaction center of the COMT enzymatic methylation reaction.

As we can see from Table 11, both of the *T̂*_3_ – (T) and (Q) components are positive, for both Loose and Normal LNO settings. These terms then combine to a (Q) – (T) correction totaling 0.08–0.09 kcal·mol^–1^, again with very pleasing agreement between the Loose and Normal LNO settings. Clearly, there are significantly more troubling sources of uncertainties in an enzyme catalysis study than a missing 0.1 kcal·mol^–1^-scale (Q) – (T) correction. Nevertheless, the option is now enabled for the first time and could be useful for reactions of species having a somewhat more complicated electronic structure. For example, barriers, open-shell, or transition metal reactions would probably have larger (Q) – (T) corrections. The additional relevance of this demonstration is that, so far, accelerated CCSDT­(Q) methods have been applied to either relatively small systems of up to 10–20 atoms or to low-dimensional systems that are the most favorable for local correlation approximations (such as linear alkane chains or water molecular clusters). This enzyme system is so far the first, very large, real-world, 3D system, where CCSDT­(Q)-quality results could be obtained (see Table S4 in the Supporting Information).

**Table 11:** Post-CCSD­(T) Reaction Energy (kcal·mol^–1^) Corrections of the COMT Enzyme Catalyzed Methylation Reaction via LNO-CCSDT­(Q)-in-LNO-CCSD­(T)/MM Calculation

	*T̂*_3_ – (T)	(Q)	(Q) – (T)
Loose	0.032	0.061	0.094
Normal	0.010	0.074	0.084

## Conclusions

We report the details of the local natural orbital (LNO) based implementation of arbitrary order CC methods in Mrcc, both for closed- and open-shell systems. Here, we focus on the LNO-based post-CCSD­(T) methods, extending our previous, highly optimized LNO-CCSD­(T) approaches. The LNO-based post-CCSD­(T) methods inherit most of the local and natural orbital approaches optimized so far for up to LNO-CCSD­(T). These include features like asymptotically linear-scaling operation cost and asymptotically constant memory and disk use, although it is very difficult to get close to the linear-scaling regime due to the large prefactor of post-CCSD­(T) methods. For further cost reduction, we make use of the point group symmetry and multilevel embedding, enabling LNO-based arbitrary order CC embedded into another LNO-CC layer and/or into DFT/MM environments.

We also report detailed accuracy and efficiency benchmarks, focusing on the practically most important LNO-CCSDT, LNO-CCSDT­(Q), and LNO-CCSDTQ methods. The LNO approximations can be flexibly tuned via a systematically converging series of predefined Loose, Normal, Tight, etc. LNO threshold sets. The reported assessments against canonical CC references for main group thermochemistry (atomization, bond saturation, barriers) and noncovalent interactions lead to the following practical conclusions:

(1) Regarding the atomic orbital basis set choice, the same recommendations can be made for LNO-based methods as for canonical post-CCSD­(T). That is, LNO approximations well preserve the intrinsic accuracy of post-CCSD­(T) methods with a given finite basis set, as demonstrated for the practical double- and triple-ζ basis sets. For CCSDTQ–CCSDT­(Q) corrections, double-ζ is excellent, while the larger effects in CCSDT and CCSDT­(Q) beyond CCSD­(T) benefit from triple-ζ basis, at least in atomizations or when a larger number of bonds are broken.
(2) The deviations due to the LNO approximations, even with Normal settings, are well below the double-ζ basis set error for all studied post-CCSD­(T) approaches. Especially for CCSDTQ–CCSDT­(Q), all LNO settings perfectly reproduce the canonical results.
(3) For the larger effects in CCSDT and CCSDT­(Q) beyond CCSD­(T), the canonical results are also reproduced to better than 90%, already with Normal settings. This translates into 0.05–0.1 kcal·mol^–1^ accuracy also in challenging bond saturation and atomization processes. This accuracy is much better than double-ζ and comparable to triple-ζ basis set incompleteness for the separate CCSDT–CCSD­(T) and CCSDT­(Q)–CCSDT effects.
(4) For the combined CCSDT­(Q)–CCSD­(T) correction, against the quadruple-ζ reference, Normal LNO deviations on the 0.01 kcal·mol^–1^ scale are 5–6 times smaller than the triple-ζ basis set error.
(5) For a small set of diatomic and triatomic molecules, the BSSE effect on LNO-(Q) contributions is generally below 0.06 kcal/mol and increases to a modest 0.2 kcal·mol^–1^ for LNO-based CCSDT–CCSD­(T). Both are fairly unaffected by the LNO approximations, indicating the sufficiency of Normal settings also from this perspective.
(6) For noncovalent interactions, the size of CCSDT­(Q)–CCSD­(T), CCSDT­(Q)–CCSDT, and CCSDT–CCSD­(T) effects, as well as the LNO errors upon threshold tightening decrease reliably to the 0.01–0.02 kcal·mol^–1^ scale. Unlike for the other tests, the cancellation of both the CCSDT­(Q)–CCSDT and CCSDT–CCSD­(T) effects, as well as the LNO errors, is highly systematic. Therefore, due to the small post-CCSD­(T) effects, Loose settings reaching 0.02 kcal·mol^–1^ RMSD are only good for qualitative assessment, and Normal LNO errors can still be comparable to the size of the post-CCSD­(T) corrections.
(7) When needed, switching to Tight or better LNO settings generally and reliably decreases the LNO errors even for atomizations, where LNO error compensations are the least possible between the reactant and product states. Due to the small post-CCSD­(T) effects for noncovalent interactions, Tight LNO may find its use there, when aiming at accuracy in both the relative and the absolute sense.
(8) While only moderate-sized alkanes up to heptane are assessed in the scaling study, the effective cost scaling is already reduced to *O*(*N* ^3^) and *O*(*N* ^4^) for LNO-CCSDT and LNO-CCSDT­(Q), respectively. Regarding parallel scaling, canonical (Q) and LNO-(Q) exhibit near-perfect parallelization with up to at least 16 threads, while the scalability reaches a saturation point more quickly for canonical CCSDT and LNO-CCSDT.

All in all, especially due to their much-reduced memory and disk requirements, LNO-CC methods extend the reach of post-CCSD­(T) accuracy well beyond that of canonical methods. For very small systems and basis sets, especially with high point group symmetry, canonical CCSDT, CCSDT­(Q), and CCSDTQ remain more efficient. For larger systems above ca. 100 orbitals, LNO-based post-CCSD­(T) methods are the most efficient for thermochemistry and noncovalent interactions. In this regime of large systems, we are confident that they will become invaluable tools to the scientific community. For example, we go beyond simple linear chain and molecular cluster applications and, in combination with multilevel correlation embedding, report LNO-based CCSDT­(Q)–CCSD­(T) correction for a real-world enzyme reaction step. This opens the door to surpassing CCSD­(T) accuracy for large, practically relevant chemical processes with accessible computational cost.

## Supplementary Material



## Notes

Electronic energies of the studied species (XLS)
